# Examining the feasibility of an economic analysis of dyadic developmental psychotherapy for children with maltreatment associated psychiatric problems in the United Kingdom

**DOI:** 10.1186/s12888-014-0346-0

**Published:** 2014-12-10

**Authors:** Nicole RS Boyer, Kathleen A Boyd, Fiona Turner-Halliday, Nicholas Watson, Helen Minnis

**Affiliations:** Health Economics & Health Technology Assessment, Institute of Health & Wellbeing, University of Glasgow, Glasgow, UK; Mental Health & Wellbeing, Institute of Health & Wellbeing, University of Glasgow, Royal Hospital for Sick Children, Glasgow, UK; Sociology, Institute of Health & Wellbeing, University of Glasgow, Glasgow, UK

## Abstract

**Background:**

Children with maltreatment associated psychiatric problems are at increased risk of developing behavioural or mental health disorders. Dyadic Developmental Psychotherapy (DDP) was proposed as treatment for children with maltreatment histories in the USA, however, being new to the UK little is known of its effectiveness or cost-effectiveness. As part of an exploratory study, this paper explores the feasibility of undertaking economic analysis of DDP in the UK.

**Methods:**

Feasibility for economic analysis was determined by ensuring such analysis could meet key criteria for economic evaluation. Phone interviews were conducted with professionals (therapists trained and accredited or in the process of becoming accredited DDP practitioners).

Three models were developed to represent alternative methods of DDP service delivery. Once appropriate comparators were determined, economic scenarios were constructed. Cost analyses were undertaken from a societal perspective. Finally, appropriate outcome measurement was explored through clinical opinion, literature and further discussions with clinical experts.

**Results:**

Three DDP models were constructed: DDP Full-Basic, DDP Home-Based and DDP Long-Term. Two potential comparator interventions were identified and defined as Consultation with Carers and Individual Psychotherapy. Costs of intervention completion per case were estimated to be: £6,700 (DDP Full-Basic), £7,100 (Consultations with Carers), £7,200 (DDP Home-Based), £11,400 (Individual Psychotherapy) and £14,500 (DDP Long-Term). None of the models of service delivery were found to currently measure effectiveness consistently. The Strengths and Difficulties Questionnaire (SDQ) was deemed an appropriate primary outcome measure, however, it does not cover all disorders DDP intends to treat and the SDQ is not a direct measure of health gain. Inclusion of quality of life measurement is required for comprehensive economic analysis.

**Conclusions:**

Economic analysis of DDP in the UK is feasible if vital next steps are taken to measure intervention outcomes consistently, ideally with a quality of life measurement. An economic analysis using the models constructed could determine the potential cost-effectiveness of DDP in the UK and identify the most efficient mode of service delivery.

**Electronic supplementary material:**

The online version of this article (doi:10.1186/s12888-014-0346-0) contains supplementary material, which is available to authorized users.

## Background

Children who experience adverse care in their early years such as maltreatment, neglect or institutional deprivation have an increased risk of developing attachment, behavioural and mental health disorders [1–3]. These disorders include: conduct disorder, attention deficit hyperactivity disorder and reactive attachment disorder [1–3]. The symptoms of these disorders often overlap, therefore for the purposes of this manuscript, we describe these as ‘maltreatment associated psychiatric problems’ (MAPP).

Children with MAPP suffer from behaviour problems that severely disrupt everyday functioning [4]. They are prone to destructive antisocial behaviour which can present as: aggression, defiance, hostility, disobedience, restlessness, anxiety and violence [5]. In addition to mental health problems, consequences of institutional deprivation and child maltreatment can impact on later adulthood social and economic functioning, well-being, criminal activity and even result in premature mortality [6–9].

A recent study by Brown et al. [10] found that people with six or more adverse childhood experiences had a 20-year reduction in life expectancy compared to those without adverse childhood experiences. In addition, economic costs of child maltreatment are high. In the United States of America (USA) the total lifetime societal cost of child maltreatment per victim was estimated to be $210,012, price year 2010 [11]. This incorporates short and long-term medical costs, losses of productivity, criminal justice and special education needs [11]. In the United Kingdom (UK), Scott et al. [12] found at age 28 public service costs for individuals with conduct disorder in childhood were 10 times higher than those who had no problems.

Despite these statistics, there have been few empirically evaluated interventions to treat psychiatric problems that arise from early trauma or neglect [13–16]. Reasons for this are due to the complexities surrounding maltreatment and psychological disorders [14] and difficulty in demonstrating intervention effectiveness and cost-effectiveness [16]. These are some of the most vulnerable children in society and effective treatment for MAPP is critically needed.

**Figure 1:**
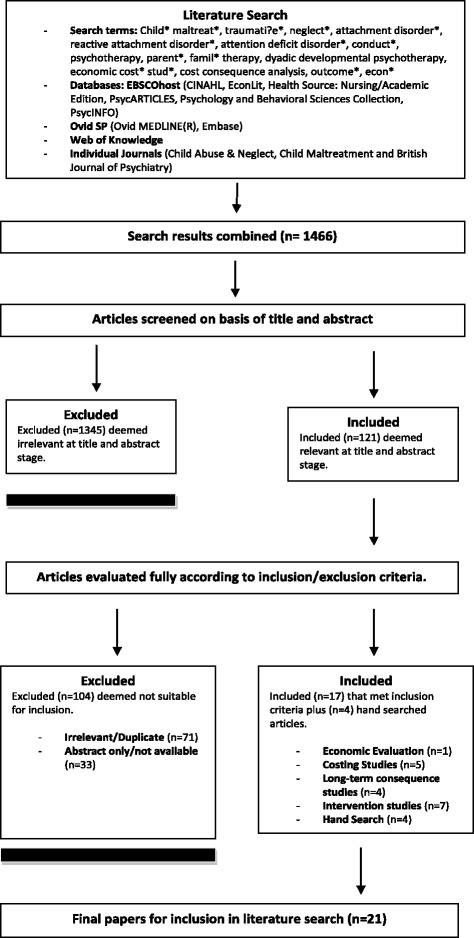
**PRISMA style flow diagram depicting details of search strategy (Moher et al. BMJ 2009; 339:b2700).**

To establish what treatments and level of evidence currently exists, a literature review on interventions for treating children with MAPP was conducted. Details of the search strategy are depicted in Figure 1. Nine databases were searched from the year 1991 to May 2012, limited to the English language and humans. The databases CINAHL, EconLit, Health Source: Nursing/Academic Edition, PsycARTICLES, Psychology and Behavioral Sciences Collection, PsycINFO), Ovid MEDLINE(R), Embase and Web of Knowledge were searched. Additionally, hand searches were conducted in journals such as: Child Abuse & Neglect, Child Maltreatment, The British Journal of Psychiatry and Cochrane Library publications. Finally, the Personal Social Services Research Unit (PSSRU) website was also hand searched for relevant publications, as the unit regularly undertakes economic work in social care which may be relevant to the literature search.

Specific searches were constructed for the population (children with attachment, conduct, attention deficit hyperactivity and reactive attachment disorders related to child maltreatment) and the intervention of interest (Dyadic Developmental Psychotherapy (DDP) and other interventions for MAPP) using keywords and subject headings. These searches were then combined with key economics terms. A total of 21 articles were included for literature review. For further details of the search strategy and the combination of specific search terms used in each database see Additional file 1.

A gap in the literature was identified regarding effective treatment for MAPP with a lack of concrete evidence. Most research focuses on effectiveness of parent training programmes for conduct disorder only, while there is little research on treatments for other maltreatment-associated problems such as reactive attachment disorder. Due to the limited evidence-base on effective treatments for MAPP, a variety of interventions comprise current practice with seemingly little effect as demonstrated by Becker-Weidman and Hughes [4].

The mellow parenting programme has shown effectiveness in improving psychosocial function of vulnerable babies and preschool children [17]. Mellow parenting is an intensive programme for families with severe relationship problems. It requires one full day of therapeutic group and family sessions per week for 14 weeks. However, when this programme was applied to school-age children with reactive attachment disorder, no measurable effects on symptoms of the disorder or on parent–child interaction were found [17]. The logical next step would be to try an even more intense treatment or therapy – especially one with a focus on the child as well as parents.

The literature highlighted that DDP could potentially be an effective treatment for MAPP. DDP is a relatively novel treatment for children who have experienced early maltreatment or neglect and now reside in foster or adoptive care [4,18]. It is a family-based intervention that focuses on building relationships between children with MAPP and their new adoptive or foster carers. The therapy helps carers to understand their child’s attachment needs, and helps the child communicate their needs and come to their carer when in distress. A DDP pilot study from the USA reported a large effect size for DDP as treatment for children with relationship-based mental health problems [4]. It compared children diagnosed with reactive attachment disorder receiving DDP to a matched control group of similar children who received a variety of other treatments typical to their condition at one [18] and four year follow-up [4]. DDP is a resource intense therapy and therefore likely to be costly to deliver, however, if it is found more effective than existing treatments, then it is possible that DDP could be a cost-effective treatment for MAPP.

Following these promising findings in the USA, DDP has since been introduced in some areas of the UK. The National Health Service (NHS) is the publicly funded healthcare system in the UK, funded primarily through taxes and providing comprehensive healthcare to all UK residents; most of which is free at the point of use. A collectively financed healthcare system such as the NHS cannot afford to fund every new clinical intervention, therefore choices need to be made about funding allocation [19], basing decisions on the incremental costs and incremental benefits of new interventions. The National Institute for Health and Care Excellence (NICE) was established to provide guidance for the most effective ways to diagnose, treat and prevent diseases and ill health [20]. Guidance is based on clinical and economic evidence to support informed decisions for funding allocation of new and existing treatments [19]. Specifically, cost-utility analysis along with demonstrated clinical effectiveness is an integral and required part of health technology assessment [19] which is the evaluation of new and existing medical technologies in comparison to current standard of care [20,21].

Before widespread implementation of DDP can occur in the NHS, both clinical effectiveness and cost-effectiveness should be established [21]. The practice and development of DDP in the UK is still in its infancy and there are plans to develop a randomised controlled trial (RCT) to determine effectiveness of DDP in a UK setting. Therefore this study was undertaken alongside a qualitative exploration of current practice of DDP in the UK [22] to determine the feasibility for an economic analysis of DDP and to discuss appropriate comparator(s), outcome measurement and service delivery models of DDP.

## Methods

### Approval

No ethical approval was required for this project as data came from a retrospective review of related published material or clinical opinion of professionals. All published material was adequately referenced so no permissions were needed. Prior to conducting interviews, the need for ethical approval was checked with NHS Greater Glasgow and Clyde Research and Development Department who triage all pending ethics applications. They confirmed that ethical approval was not required for this study because the only participants were professionals and the study would be considered audit/service evaluation. The NHS Health Research Authority does not require ethical review of studies which are considered to be solely audit or service/therapy evaluation [23]. Audiotaped verbal consent to take part in interviews was obtained from all interviewees and the transcripts were annonymised.

### Design

DDP is a complex intervention made up of many components that act independently and interdependently, making it difficult to isolate the important ‘active ingredients’ or components of the intervention [24,25]. This also makes replication of DDP difficult as DDP is introduced in different parts of the UK; possibly resulting in different modes of service delivery.

The feasibility for an economic analysis was determined by ensuring that such an analysis could meet key criteria, as defined by Drummond et al. [26], and also stipulated more recently by the Medical Research Council (MRC) in their guidance for developing and evaluating complex interventions [27]. An economic analysis must consider both costs and outcomes, as well as be undertaken in comparison to a relevant alternative.

Utilising this guidance, four key points were established that would need to be addressed for economic evaluation of DDP in the UK. This paper sought to: (i) establish the core model(s) of service delivery for DDP by appropriately defining the intervention, (ii) determine an appropriate comparator intervention(s), (iii) explore likely costs of DDP models and comparator interventions, and (iv) explore an appropriate primary outcome measure for determining the effectiveness of DDP. Economic evaluations are often costly; therefore the decision to undertake evaluation should be well informed by economic considerations such as those listed above [27].

A societal perspective was deemed appropriate for exploring the feasibility for an economic analysis so that all potential costs and benefits were considered [26–28].

### Data collection

This study fed into and utilised data from a parent qualitative study on DDP delivery in the UK [22]. DDP therapists were contacted throughout the UK and asked to participate in qualitative phone interviews. Health economics questions to explore DDP resource use, relevant outcome measurement and comparator(s) were incorporated into the semi-structured interview schedule. Phone interviews were then conducted with 13 DDP therapists, based in eight UK sites. All interviews took place between March and May 2012 and were recorded, transcribed and anonymised by omitting names of interviewees from transcripts. Phone interviews were followed up with questionnaires to garner additional information regarding resource use in service delivery, opinions on comparator interventions and on appropriate outcome measurement. These questionnaires were sent to DDP therapists who had previously been interviewed as well as additional therapists who attended a national DDP conference. Once relevant comparator interventions emerged, separate questionnaires specific to these interventions’ resource use were devised and emailed to experts. Further details of the data collection methods are published elsewhere [22].

### Analysis

Thirteen DDP therapists, located across all four countries of the UK, consented to take part in phone interviews. Data were transcribed and used as a data input to develop models of DDP service delivery. Each transcript was thoroughly examined for any relevant health economics information, using the four key points as a framework for reference.

Using the information from the transcripts, three alternative DDP delivery models were constructed to broadly represent the alternative delivery methods, resources used and practices of DDP throughout the UK. Two relevant comparator interventions emerged from the data; however there was no resource use data from the transcripts for these comparators. Therefore, clinical experts were contacted and resource use data elicited in questionnaires in order to construct general models of service delivery for the two possible comparators.

A cost analysis was undertaken for the models of DDP and comparator interventions. Resource use estimates were combined with unit costs to determine the costs to the NHS, families and social services. NHS costs included time spent preparing, conducting and reviewing DDP or comparator sessions, and materials. Costs to families included time away from work needed for carers and child to participate in the therapy as well as travel expenses. Travel expenses to participate in therapy were a social services cost except when therapy was home-based and was then considered an NHS cost.

All costs were reported in 2011 UK Pounds Sterling. Unit costs were extracted from the PSSRU Unit Costs 2011 [29] for NHS cost categories. Time off work for primary and secondary carers were taken respectively from Her Majesty’s Revenue and Customs minimum wage guidance [30] and the Office for National Statistics April 2011 median wage [31]. Travel expenses were utilised from the Department for Transport policy guidance website [32]. Costs which were accrued over one year were discounted at 3.5% as recommended by NICE [21]. Discounting reduces future costs and benefits primarily due to time preference, a concept that most people would prefer to have money now versus later on in the future [26]. Discounting is therefore employed to obtain the ‘present value’ of future costs or effects.

A range of possible outcome measurements for DDP suggested in interview transcripts was recorded and tabulated. These relevant outcome measures were explored for suitability using evidence in literature then discussed among clinical experts. The advantages and disadvantages of each possible measure were examined in order to establish the most appropriate outcome measures from a clinical effectiveness standpoint, and then considered for appropriateness from an economic analysis standpoint.

## Results

### Defining the intervention

DDP practice and service delivery was found to vary greatly throughout the UK. Therefore three core models of DDP were constructed to broadly represent current service delivery in the UK: DDP Full-Basic, DDP Home-Based and DDP Long-Term. These three models were considered ‘full’ versions of DDP where DDP was used as a therapeutic approach with child and carers together and often with separate work done with carers alone [22]. A distinct, ‘lighter’ approach was found to be practiced when DDP principles of Playfulness, Acceptance, Curiosity and Empathy (PACE) [33] were used to structure conversations, training and work with carers [22]. Differences exist in service delivery between the UK and original model developed by Dan Hughes [34] and piloted by Arthur Becker-Weidman [18] in the USA. In Becker-Weidman et al.’s [18] pilot study, 23 two-hour sessions over 11 months were the average service delivery requirement of DDP. Carer-only sessions (without the child) took place before and after family sessions, while the child waited in a separate room. Such an approach would not be seen as acceptable the UK (unpublished observations: Quilter, MT; Follan, M; Blower, A; and Minnis, H). The transcripts revealed that in the UK, carer-only sessions tend to take place over the phone, email or on a separate occasion.

### DDP full-basic

This model is termed ‘Full-Basic’ as it represents the full DDP service delivery model with the fewest total sessions and shortest duration per session. This model has an average duration of four months, in which each case will receive 15 family sessions lasting one hour and including both carers and child. Twelve additional sessions take place with carers only lasting 30 minutes each. This model is based on a practice whereby 30% of family sessions take place with two therapists. The model reflected this increase in therapist time as an increased number of family sessions (i.e. 20 sessions, rather than 15) to account for additional NHS provider time. The facilities in this model are most compatible with the original DDP model [33,34]. The therapeutic space has video recording/playback for review after sessions and sofas and toys to make the setting comfortable for the entire family. In this ‘Full-Basic’ model of service delivery, an average of 12 cases per year would be seen per therapist.

### DDP home-based

This model of DDP delivery varied considerably from the USA model in that the service is delivered in the family’s home. A DDP therapist will travel to each family home, seeing each case for approximately 14 months, consisting of 28 family sessions and a further 28 sessions with carers only. In this model therapists arrive at the carers’ home 45 minutes before the child comes home from school to set up video equipment and meet with carers. The family session would then take place and be recorded for another 45 minutes. In this ‘Home-Based’ model of service delivery, an average of 6 cases per year would be seen per therapist. It is important to note that in this model, some resources are transferred between the NHS and families. There is less of a travel burden required for families travelling to NHS facilities. NHS travel expenses increase; however, there will be lower costs to the NHS as some of the overheads for facilities are transferred to the families, possibly freeing up therapeutic space for other NHS interventions.

### DDP long-term

This model represents the most resource intense delivery in terms of number and duration of sessions. Each case lasts for one year, with an average of 19 family sessions lasting one hour and 45 minutes each. In addition, 24 sessions lasting one hour and 30 minutes take place with carers only. Due to the intensity of delivery of this model, a DDP therapist would only see approximately four cases per year. With regards to therapeutic space, no video recording, sofas or toys are provided in addition to what is already available in a standard therapy clinical space. This aspect again diverges from the original model developed and delivered in the USA [18].

### Resource use and unit costs

Costs were attributed to three main areas: NHS, families and social services. The resource use in the NHS consisted of professional time which was used for: consultations with carers only, consultations with families, preparation and review of the case and peer supervision. DDP needs to be provided by a trained clinical psychologist (NHS staff salary Band 8) [35]. DDP is also resource intensive in terms of carers time, which may involve time off work for one or both carers; a cost to families. Additionally, in the Home-Based model, use of the carers’ home was a resource burden that fell on the family. In the Full-Basic and Long-Term models, the time and expense for carers travelling to the clinic is also considered. Carers are able to claim reimbursements from social services for travel expenses if they choose. Table 1 details the resource use and costs for each of the three DDP models. The unit cost for a clinical psychologist was £135 per hour [29]. This is a societal cost including wages, salary on-costs (employer incurred national insurance and pensions contributions), overheads, capital overheads, costs to the provider for office, telephone, education and training, supplies and services, and utilities such as water, gas and electricity [29]. For the DDP Home-Based model, the non-societal unit cost for a clinical psychologist was used (£60 per hour [29]) as overheads and use of a separate clinical space were not costs associated with use of an NHS facility.

**Table 1:** **Resource use, unit cost and cost per case for DDP models**

			**DDP Full-Basic**				**DDP Home-Base**					**DDP Long-Term**			
**Area**	**Cost item**	**Provider**	**Number of sessions**	**Duration (hours)**	**Unit cost (£)**	**Cost/Case (£)**	**Number of sessions**	**Duration (hours)**	**Unit cost or range (£)**	**Cost/Case (£)**	**Discounted (£)**	**Number of sessions**	**Duration (hours)**	**Unit cost (£)**	**Cost/Case (£)**
NHS	Consultation with carer	Clinical Psychologist Band 8	12	1	135	810	28	1	60	1,260	1,253	24	2	135	4,860
	Consultation with carer and child	Clinical Psychologist Band 8	20	1	135	2,700	28	1	60	1,260	1,253	19	2	135	4,489
	Prep time/Review time	Clinical Psychologist Band 8	20	1	135	1,350	28	0	60	560	557	24	1	135	1,620
	Peer Supervision	Clinical Psychologist Band 8	4	2	135	810	-	-	-	-	-	4	2	135	1,080
	Video Recording Equipment	-	-	-	-	19	-	-	156	156	155	-	-	-	-
	Sofa	-	-	-	-	9	-	-	-	-	-	-	-	-	-
	Toys	-	-	-	-	4	-	-	-	-	-	-	-	-	-
	Travel expense	Clinical Psychologist Band 8	-	-	-	-									
**TOTAL NHS**						**5,702**				**6,036**	**6,001**				**12,049**
Family	Time off work/other duties: Carer 1	-	15	3	6	228	28	2	6	255	254	24	4	6	620
	Time off work/other duties: Carer 2	-	15	3	3	634	28	2	17	711	706	24	4	17	1,726
	Sofa	-	-	-	-	-	-	-	0	0	0	-	-	-	-
	Toys	-	-	-	-	-	-	-	0	0	0	-	-	-	-
	Use of home for session	-	-	-	-	-	28	2	5	140	139	-	-	-	-
**TOTAL FAMILY**						**862**				**1,106**	**1,100**				**2,346**
Social Services	Travel expense		30	12 miles	0.4/mile	144	-	-	-	-	-	48	5 miles	0.4/mile	96
**TOTAL SOCIAL SERVICES**						**144**					-				**96**
**TOTAL COSTS**					**TOTAL**	**6,708**			**TOTAL**	**7,141**	**7,101**			**TOTAL**	**14,490**

Unit costs for materials were taken from market prices and estimates for total years of use were applied and discounted to present value. The discounted price of materials was then divided by the total number of cases seen per year by each DDP model.

As DDP is intended for foster or adopted children, it was assumed at least one caregiver would look after the child full-time. Full-time foster carers in the UK receive the minimum wage and, therefore, foster carers’ time spent in sessions was reimbursed at that wage [30]. The second carer was assumed to work full time, and therefore the average wage in the UK was used to reflect the opportunity cost of their time away from work [31]. Use of the home for sessions in the Home-Based model was accounted for by a £5 ‘token’ cost to the family to reflect the opportunity cost of using their facilities. This token cost was considered appropriate because even though the facility cost was transferred from the NHS to the family in this model, the family would not incur heavy NHS overheads, but was assumed to still keep ‘the lights on’ regardless of a DDP session taking place or not.

For travel expenses, transport was assumed to be by car and a £0.40/mile reimbursement rate was applied as per Department for Transport policy guidance [32]. The DDP Home-Based model assumed a mean travel cost per case of £200 per month based on clinical opinion. Travel expenses in the DDP Full-Basic and DDP Long-Term models assumed an average mileage that covered the service area which would also be reimbursed by social services.

### Appropriate comparators

Comparator interventions that were mentioned by experts included: Individual Psychotherapy (IP), Consultation with Carers (CwC), Cognitive Behavioural Therapy, Play Therapy, Multi-Dimension Treatment Foster Care and many others. There was however, little agreement between experts regarding which therapies should be offered to which children [22] and none of these therapies were available in all areas. In the absence of DDP, children with MAPP in the UK could therefore be treated with a wide variety of interventions. This resulted in the decision that, if a future trial were undertaken, Child and Adolescent Mental Health Services (CAMHS) and services-as-usual (which include CwC and IP interventions) would be the most likely comparator as there is no ‘gold standard’ treatment used in every area. As CwC and IP are the most common standardised interventions in current CAMHS practice, these were used in this study as exemplars for the evaluation of control interventions in any future trial.

### Consultation with carers

The CwC model consists of 10 consultation sessions lasting one and a half hours where a clinical professional such as a clinical nurse specialist or clinical psychologist (NHS staff salary Band 8) [35] advises carers on issues they may be having with their children and give parenting advice focused towards children with mental health problems. Consultations are complete in 10 months and each specialist can see approximately 24 cases per year. Approximately one out of 10 of these sessions take place at the carers’ home. Ideally both carers participate in sessions, but in reality both carers are present about half of the time and this is reflected in the model.

### Individual psychotherapy

IP is a resource intensive intervention. Therapy involves 60 sessions over two years with each therapist seeing approximately eight cases per year. Each session lasts 50 minutes and 30 minutes are needed for preparation and review of the session. The health professional delivering psychotherapy would be an experienced clinical psychologist (NHS staff salary Band 8) [35]. Toys are the only material item provided and were estimated to cost £25 per year per family.

A carer does not take part in IP, but their time is still needed to drop the child off and wait to pick them up. Therefore, their time driving to and from (one hour assumed) plus the length of the session is a cost to the family in terms of time away from work or other activities. Table 2 details resource use and costs for the two comparator interventions. Similar assumptions made in the DDP models such as travel time, mode of travel and discounting if the intervention lasts over one year, were applied to comparator models except for the unit cost for toys which was given. Table 3 summarises total costs to NHS, families and social services of the five different models.

**Table 2:** **Resource use, unit costs and cost per case for comparator models**

			**Consultation with carers**				**Individual psychotherapy**				
**Area**	**Cost item**	**Provider**	**Number of sessions**	**Duration (hours)**	**Unit cost (£)**	**Cost/Case (£)**	**Number of sessions**	**Duration (hours)**	**Unit cost (£)**	**Cost/Case (£)**	**Discounting (£)**
NHS	Consultation with child	Clinical Psychologist Band 7.5	9	2	113	1526	-	-	-	-	-
	Consultation with child	Child Psychotherapist Band 8	-	-	-	-	60	1	135	6750	6522
	Consultation with carers in home	Clinical Psychologist Band 7.5	1	2	57	85	-	-	-	-	-
	Prep/Review time	Clinical Psychologist Band 7.5	30	2	113	5085	-	-	-	-	-
	Prep/Review time	Child Psychotherapist Band 8	-	-	-	-	60	1	135	4050	3913
	Toys	-	-	-	-	-	60	1	-	50	48
**TOTAL NHS**						**6695**				**10850**	**10483**
Family	Time off work/other duties: Carer 1	-	10	3	6	152	60	2	6	669	646
	Time off work/other duties: Carer 2	-	5	3	17	211	-	-	-	-	-
	Use of home for session	-	1	2	5	5	-	-	-	-	-
**TOTAL FAMILY**						**368**				**669**	**646**
Social Services	Travel expense		18	5 miles	0.4/mile	36	120	5 miles	0.4/mile	240	232
**TOTAL SOCIAL SERVICES**						**36**				**240**	**232**
**TOTAL COSTS**					**TOTAL**	**7100**			**TOTAL**	**TOTAL 11759**	**11361**

**Table 3:** **Total cost per case of the 5 alternative models**

	**DDP Full-Basic**	**DDP Home-Based**	**DDP Long-Term**	**Consultation with carers**	**Individual psychotherapy**
NHS	£ 5,702.01	£ 6,001.08	£ 12,048.75	£ 6,695.25	£ 10,483.09
Family	£ 862.38	£ 1,210.04	£ 2,345.68	£ 368.46	£ 646.18
Social Services	£ 144.00	-	£ 96.00	£ 36.00	£ 231.88
Total Costs	£ 6,708.39	£ 7,211.12	£ 14,490.43	£ 7,099.71	£ 11,361.15

Total cost per case for all models ranked from lowest to highest are: DDP Full-Basic (£6,700), CwC (£7,100), DDP Home-Based (£7,200), IP (£11,400) and DDP Long-Term (£14,500).

The least expensive treatment per case was DDP Full-Basic, with the fewest sessions and the shortest session duration. CwC was the second least costly treatment model, however, it should be noted that the time required for preparation and review of each case takes three times as long as providing the actual sessions. IP costs considerably more than CwC and the Full-Basic and Home-Based DDP models. The main cost driver is the large number of sessions, on average 60 per case. The most expensive treatment was DDP Long-Term due to its increased number and longer duration of sessions. This model was also the most costly to families as it required the most time from carers, representing a large opportunity cost of their time.

### Appropriate principal outcome measure

It was evident from the interview transcripts that few DDP practices consistently measured outcomes of their cases; and some did not consider evaluation of their cases or the ‘impact’ of DDP at all. When prompted, clinical opinion on appropriate outcome measures for DDP and its comparators varied greatly amongst the DDP therapists interviewed. The most common outcome measures suggested in interviews were the Strengths and Difficulties Questionnaire (SDQ) [36] followed by the Kim Golding Carer Questionnaire. The later does not have any population-based validity; however it was developed specifically for DDP to address MAPP and behaviours that DDP tries to remedy. In the literature, the Child Behaviour Check List (CBCL) and Rutter Questionnaire are commonly cited. The CBCL was used in the Becker-Weidman [4] pilot study, however the SDQ was developed to cover the same domains as the CBCL and Rutter, and is now by far the most commonly used internationally. It was also directly validated against the Rutter Questionnaire [37].

The SDQ is well validated and available in 50 different languages. It is completed by carers or teachers for children aged 4–17 or by self-completion of children aged 11–17 years. It has 25 items divided into five symptom scales making up positive and negative attributes which are: emotional symptoms, conduct problems, hyperactivity, peer relationships and prosocial behaviour [37]. The symptom scales are then summed (not including prosocial scale) to make up a Total Difficulties score. The SDQ is a measure of mental health and can be used to measure change as a result of treatment, however, it does not measure reactive attachment disorder. The Relationship Problems Questionnaire addresses this problem and is well validated. This questionnaire however has been used previously in a small-scale audit of DDP and was found not to be responsive to change (personal communication, May 2010, Julie Hudson).

Given the information from the interview transcripts and outcomes used in similar studies to date, it seems that the SDQ is likely to be an appropriate outcome measure for evaluating DDP in an RCT. The SDQ could be appropriate for determining short-term effectiveness in terms of mental health, however in terms of an economic analysis; a longer-term health gain is required. The short-term cost-effectiveness of DDP could be calculated by determining the incremental cost per improvement on the SDQ scale (improvement in mental health); however, a longer-term focus is required to show wider health and economic gains. Quality-adjusted life years (QALY) are the reference case outcome preferred by NICE for demonstrating the cost-effectiveness in health technology assessment and also for public health interventions [21,38]. QALYs consider the life expectancy impacts of health care interventions and adjust any life year gains by the quality of that life. To calculate QALYs a preference based utility measure is needed; however, currently there are few validated preference based utility measures for children. The Child Health Utility 9D (CHU9D) measure of health related quality of life may be a suitable option. It is a generic paediatric preference based outcome measure for children ages 7–17 years and can be used for QALY calculation [39]. Therefore it would be suitable for the majority of children in the age range for DDP treatment. CHU9D is a self-completion questionnaire, and could be easily incorporated into any future trial of DDP as a secondary outcome measure.

## Discussion

### Clearly defining the intervention

In an attempt to clearly define the DDP intervention as practiced in the UK, three of the most distinct practices were modelled for comparison, demonstrating differences in DDP service delivery. DDP Full-Basic is the most standardised model of DDP and is the most compatible with the original USA based model [18,34]. DDP Full-Basic has the lowest costs per family and its standardisation may contribute to that. The model’s relatively short completion time and video recording equipment may also prove more effective as Bakermans-Kranenburg, et al.’s [40] meta-analysis on attachment interventions (70 studies included) found that interventions with more than 16 sessions were less effective than those with fewer sessions, although the studies concerned were with younger children who had less serious problems. The most effective interventions had a clear behavioural focus and video feedback [40]. This type of model (DDP Full-Basic) would be pursued in designing a future RCT trial of DDP for these reasons stated above and because current practice reflects DDP Full-Basic in UK sites ready and willing to host a trial of DDP.

### Appropriate choice of comparator

In the UK, children with MAPP can be treated with a wide variety of interventions which vary from area to area. In the accompanying paper Turner-Halliday et al. [22] found child and adolescent mental health services (CAMHS) varied throughout the UK in terms of how comparator interventions were utilised, with no common ‘gold standard’ intervention. Therefore, if an exploratory trial were to take place with child and adolescent mental health services acting as the general comparator, it would be important to make a distinction between the different interventions used by CAMHS rather than combining them into one category. In the accompanying paper Turner-Halliday et al. [22] identified that CwC and IP are the most common standardised interventions currently used by CAMHS (current practice), and therefore these two interventions were modelled in this study as exemplars for the evaluation of control interventions in any future trial. We estimated the costs involved in IP and CwC as these were the most likely comparator interventions for DDP.

### Exploring likely costs of DDP and comparator interventions

The total cost per case for each intervention varied from the least costly at approximately £6700 (DDP Full-Basic) to the most costly at approximately £14,500 (DDP Long-Term).

When specifically looking at costs to the NHS and social services, two DDP models (Full-Basic and Home-Based) were less costly than the two comparators. This is noteworthy as the majority of the cost burden for each model falls on the NHS. If DDP proves to be an effective intervention, the NHS could potentially reduce costs while offering more effective treatment: a very attractive strategy in both service delivery and economic terms. DDP Long-Term in contrast, was the most costly intervention totalling just over £12,000 per case to the NHS. Therefore, we would consider this mode of DDP delivery to be inappropriate to test in an RCT framework. Costs to families differed between DDP and comparators; the latter being less costly. All models of DDP placed a higher burden on families in terms of time away from work or other activities in order to participate in DDP sessions. Involving carers in therapy is a key aspect of DDP that may act as the ‘active ingredient’ of its effectiveness. It is nonetheless an extra time burden to place on families. Costs to social services depended on the travel burden placed on each model assuming travel would be by car and expenses claimed. Therefore, the interventions that required the least travel (or the least amount of sessions) were least burdensome to social services. This is the only sector where DDP Long-Term is not the most costly model: costs of IP exceed it due to the high number of sessions requiring increased travel over a two year period.

This cost analysis was an important component of the feasibility study, in terms of understanding the likely costs of the alternative models of DDP and comparators. However, it is only a partial evaluation and cannot inform decision-making regarding which alternative intervention is efficient. For that we need information about both the costs and outcomes of the different models of DDP and comparator interventions. Further research is needed to determine the most cost-effective intervention for treating MAPP.

### Primary outcome measure

To date no single outcome measure has been collected consistently for the DDP models and the two comparator interventions, therefore we cannot comment on the effectiveness of each of the alternative interventions. The next step would be an exploratory trial in which appropriate outcome measures could be investigated while gathering effectiveness data. A full economic analysis could be used to explore the cost-effectiveness of the various models of DDP service delivery and comparator interventions, and it would ideally be performed alongside a clinical effectiveness trial of DDP.

The interview transcripts [22] suggest a strong clinical preference for the SDQ as an appropriate outcome measure for DDP. The SDQ is used internationally and has been translated into over 50 different languages [36]. With experience of using the SDQ and its ease of use, it seems to be a feasible outcome measure for DDP therapists to use and gather accurate outcomes of DDP and comparators. However, the fact that Kim Golding felt the need to develop a separate outcome measure specifically for DDP suggests that the SDQ may not fully quantify outcomes of MAPP. Therefore, in any future trial of DDP, both measures might be useful to get a broad picture of potential effectiveness of DDP. It will also be crucial to include a quality of life measure such as the CHU9D that can be used to elicit QALY outcomes for an economic analysis to inform decision makers’ choices regarding allocation of healthcare resources.

### Strengths and limitations

This feasibility study is the first of its kind to define and model DDP as practiced in the UK, and consider its possible comparators and potential measures of effectiveness. This study highlighted that no consistent outcome measures are currently being collected amongst practicing DDP sites in the UK, therefore it was not possible to make effectiveness and cost-effectiveness comparisons between the DDP models and the two possible comparator models identified in this study. This limits the ability to conduct a full economic evaluation of DDP which would enable decision makers to effectively allocate resources to the treatment of MAPP. The results of this study should be taken as indicative only, as they are based on a systematic literature review, qualitative data and clinical expert opinion rather than new empirical data. The cost analysis undertaken was based on resource use estimates garnered from the interview transcripts and clinical experts, and is subject to the assumptions made for each model. The results of this study may not be generalizable internationally due to differences in health care system, delivery and funding contexts that differ markedly from the UK. This feasibility study has established that an economic analysis of DDP is feasible and can feed into the design of a future trial of DDP in a UK setting.

## Conclusions

An economic evaluation of DDP in the UK is feasible, and could be undertaken alongside a trial which could explore the feasibility of recruitment, and a variety of potential outcome measures. Such an exploratory study could also incorporate quality of life measurements, so as to improve economic relevant evidence for DDP. An economic evaluation could be conducted alongside any future study of DDP in the UK, populating the model of DDP being used with study specific resource use and outcome data to give a more precise estimate of the costs and outcomes. An identical process would need to be undertaken to estimate costs and outcomes of the comparator intervention, so that DDP can be compared incrementally to establish cost-effectiveness. Economic evaluation alongside an exploratory RCT of DDP could inform on the potential cost-effectiveness of DDP in the UK in comparison to current practice. Ideally such an economic evaluation would not only consider the short term effectiveness of DDP but also consider the longer-term economic outcomes, including quality of life that allow for QALY calculation and presentation of cost-effectiveness in terms of incremental cost per QALY gained.

## Additional file

Additional file 1:
**Details of the search strategy employed to conduct the literature review.**
