# Supplementary material for: Examining the feasibility of an economic analysis of dyadic developmental psychotherapy for children with maltreatment associated psychiatric problems in the United Kingdom
Source: BMC Psychiatry. 2014 Dec 10;14:346. doi: 10.1186/s12888-014-0346-0 (PMC4299309; doi:10.1186/s12888-014-0346-0)
Supplement: Additional file 1: — Details of the search strategy employed to conduct the literature review. [file 12888_2014_346_MOESM1_ESM.docx]

# Additional file 1

Additional file 1: Search strategy for literature review

Search terms were divided into four categories: child maltreatment and related disorders, DDP, other treatments/interventions for MAPP and economic studies. Categories and terms are listed below.

**Child maltreatment and related disorders:** Child* maltreat*, traumati?e*, neglect*, attachment disorder*, reactive attachment disorder*, attention deficit disorder*, conduct*

**DDP:** dyadic developmental psychotherapy

**Other treatments/interventions for MAPP:** psychotherapy, parent*, famil* therapy, economic cost* stud*

**Economic studies:** cost consequence analysis, outcome*and econ*.

Search terms in each category were connected with Boolean operators OR and AND giving thousands of results. Each category search was then connected with Boolean operator AND to return an initial total of 1466. Titles and abstracts were checked and 121 articles were deemed relevant to the scope of this study. Full papers were then checked and 33 articles were abstract only or unavailable and 71 papers were excluded when inclusion/exclusion criteria applied which are as follows.

Inclusion criteria:

- Primary or secondary research relating to attachment, conduct or attention deficit disorders that are related to CM, DDP, other treatment options and economic or costing studies
- English language only
- Published literature only
- 1991 onwards

Exclusion Criteria:

- Not English language
- Unpublished research, abstract only, conference proceedings
- Pre-1991
- CM not related to attachment, conduct or attention deficit disorders
- Adults over 18
- Treatment interventions not related to CM, attachment, conduct or attention deficit disorders

Hand searches of Child Abuse & Neglect, Child Maltreatment, British Journal of Psychiatry, Cochrane Library and the Personal Social Services Research Unit (PSSRU) website revealed 4 additional relevant articles bringing the total number of articles included in literature review to 21.
